# The Role of Interleukins in Recurrent Implantation Failure: A Comprehensive Review of the Literature

**DOI:** 10.3390/ijms23042198

**Published:** 2022-02-16

**Authors:** Konstantinos Pantos, Sokratis Grigoriadis, Evangelos Maziotis, Kalliopi Pistola, Paraskevi Xystra, Agni Pantou, Georgia Kokkali, Athanasios Pappas, Maria Lambropoulou, Konstantinos Sfakianoudis, Mara Simopoulou

**Affiliations:** 1 Centre for Human Reproduction, Genesis Athens Clinic, 14-16, Papanikoli, 15232 Athens, Greece; info@pantos.gr (K.P.); agni.pantos@gmail.com (A.P.); georgiakokkali@gmail.com (G.K.); a-pappas@hotmail.gr (A.P.); sfakianosc@yahoo.gr (K.S.); 2 Laboratory of Physiology, Medical School, National and Kapodistrian University of Athens, 75, Mikras Asias, 11527 Athens, Greece; sokratis-grigoriadis@hotmail.com (S.G.); vagmaziotis@gmail.com (E.M.); kalliapistola@gmail.com (K.P.); evixistra@gmail.com (P.X.); 3 Laboratory of Histology and Embryology, School of Medicine, Democritus University of Thrace, 68100 Alexandroupolis, Greece; mlambro@med.duth.gr

**Keywords:** recurrent implantation failure (RIF), in vitro fertilization (IVF), infertility, interleukins, embryo implantation, uterus, decidua

## Abstract

Recurrent implantation failure (RIF) is a multifactorial condition affecting 10–15% of in vitro fertilization (IVF) couples. Data suggest that functional dysregulation of the endometrial immune system constitutes one of the main pathophysiological mechanisms leading to RIF. The aim of this article is to provide a thorough presentation and evaluation of the role of interleukins (ILs) in the pathogenesis of RIF. A comprehensive literature screening was performed summarizing current evidence. During implantation, several classes of ILs are secreted by epithelial and stromal endometrial cells, including IL-6, IL-10, IL-12, IL-15, IL-18, and the leukemia inhibitory factor. These ILs create a perplexing network that orchestrates both proliferation and maturation of uterine natural killer cells, controls the function of regulatory T and B cells inhibiting the secretion of antifetal antibodies, and supports trophoblast invasion and decidua formation. The existing data indicate associations between ILs and RIF. The extensive analysis performed herein concludes that the dysregulation of the ILs network indeed jeopardizes implantation leading to RIF. This review further proposes a mapping of future research on how to move forward from mere associations to robust molecular data that will allow an accurate profiling of ILs in turn enabling evidence-based consultancy and decision making when addressing RIF patients.

## 1. Introduction

Successful embryo implantation constitutes a highly organized, albeit perplexing process, where the developing blastocyst interacts and adheres to the receptive endometrium known as “decidua” [1,2]. Endometrial decidualization constitutes the primary driver of a successful pregnancy and encompasses several cellular, genetic, and epigenetic modifications of the endometrium. This process takes place during the time defined from the mid-luteal phase, prior to embryo implantation, till the early stages of the pregnancy [3,4]. Briefly, decidua formation involves the morphological and functional differentiation of the human endometrial stromal cells (ESCs) as well as of the endometrial immune cells, which in turn regulate extracellular matrix remodeling, angiogenesis, foster immune tolerance, and tissue invasion [4,5]. These complex processes are mainly regulated by progesterone, which acts synergistically with cyclic adenosine monophosphate (cAMP), controlling the expression patterns of several master regulator proteins and transcription factors, including homeobox A10 (HOXA10), forkhead box O1 (FOXO1), signal transducers and activators of transcription (STAT), and heart and neural crest derivatives expressed transcript 2 (HAND2). These molecules create a perplexing network, which is mandatory for proper decidualization and thus for successful embryo implantation [4,5].

Implantation refers to a complex process where the early embryo first attaches to the surface of the differentiated endometrium, namely the decidua, and then invades the decidua epithelium in order to form the placenta [2]. Implantation occurs during a short time frame, which is widely known as the “window of implantation”. The window of implantation is the period described between days 16–22 of a 28-day menstrual cycle, approximately 5–10 days following ovulation [2,6,7]. During this period a perplexing molecular cross-talk is established between the developing embryo and the receptive endometrium, leading to embryo-maternal tolerance and finally to implantation [2,6,7]. In order to better understand implantation events, reproductive scientists divided the implantation process into three distinct stages [8]. During the first stage, the developing embryo interacts with the implantation site, which is commonly located on the upper and posterior walls of the uterus. Apposition starts approximately two to four days following morula entrance in the uterine cavity. During this time, the developing embryo reaches the blastocyst stage and hatches from the zona pellucida in order to align to the receptive endometrium. This alignment is achieved via the interactions of embryo L-selectins with the respective ligands expressed from the endometrium. The second stage involves the strong attachment of the blastocyst to the implantation site. Embryo adhesion is mediated by the actions of integrins, which are expressed from both trophoblast cells and uterine luminal epithelium. Finally, during the last stage of the implantation process, the trophoblast cells further proliferate and differentiate developing thin folds called invadopodia. These differentiated cells penetrate the endometrial epithelium and invade the underlying endometrial stroma. The final end-point of this process is spiral artery remodeling and the placenta formation [9].

Considering natural conception rates, it seems that the chance of conception per menstrual cycle is rather low, reaching 30%. This means that two-thirds of potential natural conceptions are lost due to implantation failure [2]. Similar observations are also made in the context of assisted reproduction techniques (ART) management. The probability of implantation failure is approximately 70% as deduced from the fact that the implantation rate per embryo transfer reaches 30% [10,11]. Acknowledging these data, albeit implantation failure may entail a pathological condition, at the same time it constitutes, from an evolution standpoint, a physiological “barrier” leading to the establishment of a competent pregnancy.

However, there is a special category of infertile patients presenting with an abnormally increased proportion of consecutive implantation failures in the context of in vitro fertilization (IVF) treatment, despite the transfer of good-quality embryos. This phenomenon is known as recurrent implantation failure (RIF) and affects approximately 10–15% of women undergoing IVF treatment, globally [12]. Recurrent implantation failure constitutes a multifactorial condition for which there is no consensus yet regarding its definition [11]. The controversial criteria, indicating its highly variable nature, include the number of failed IVF cycles, the number of embryos transferred as well as maternal age [10,13]. Despite the fact that several definitions have been proposed, the most widely accepted is the one described by the failure to achieve a positive pregnancy test following three or more consecutive transfers of good quality embryos [10,11,13,14,15,16]. The discrepancies regarding RIF definition have led to a lack of a standardized, efficient, and universally accepted protocol for RIF management, making RIF one of the most challenging conditions reproductive scientists should deal with in daily clinical practice [16].

With regards to the pathophysiology of RIF, several mechanisms and conditions have been proposed, including genetic, metabolic, and hormonal pathologies as well as uterine, male, and embryological abnormalities [13]. Moreover, there are several risks factors for RIF such as advanced maternal age, congenital anatomical abnormalities of the female reproductive system, lifestyle parameters (e.g., smoking), increased body mass index, and infections (e.g., chronic endometritis) [13]. However, in 30% of RIF cases, the underlying pathology remains unidentified hence described as unexplained RIF, making management of these cases even more complicated. Recently, published data studying the preconception period indicated that among unexplained RIF cases, there is a high prevalence of women presenting with endometrial immune system dysregulation [17,18,19].

In view of the association between RIF and the endometrial immune system profile, it should be highlighted that a prerequisite of paramount importance for successful implantation is the balanced activation of the endometrial immune system for both semi-allogenic embryo acceptance as well as for implantation per se [20]. Embryo–maternal communication is principally mediated via the action of several cytokines and chemokines and especially via interleukins (ILs), which constitute an essential part of the uterine microenvironment [21,22]. Interleukins significantly affect the process of embryo implantation, from decidua formation and embryo acceptance to trophoblast invasion and placenta formation. However, abnormal IL production may detrimentally affect implantation, despite embryos being of good quality and of high developmental dynamic, subsequently leading to RIF [19,20,23,24,25,26,27,28,29]. This abnormal endometrial immune profile is characterized by the presence of high levels of pro-inflammatory cytokines, originating from the impaired balance of abnormal high cytotoxic immune cell subtypes and decreased regulatory cellular elements [17,18,19]. The impaired cytotoxic endometrial immune microenvironment triggers molecular events mimicking “alloimmune graft rejection”, subsequently leading to embryo rejection and thus to pregnancy failure [17,18,19]. However, the exact role of ILs on the pathophysiology of RIF is poorly understood and still debatable [20,25,29].

Acknowledging the aforementioned, it is of paramount importance for the assisted reproduction scientific community to better understand the complex molecular and cellular mechanisms regulating ILs’ actions during embryo implantation and pregnancy establishment, in order to shed light on the exact role of these molecules on the pathophysiological basis of RIF. Considering the discrepancies observed in the literature ranging from understanding to managing RIF, this review aims to provide a collective summary, and an in-depth evaluation of the current knowledge contributed from basic research studies on the molecular and cellular actions of ILs during embryo implantation, especially highlighting the disrupted molecular pathways observed in women presenting with RIF. Moreover, authors comprehensively present and analyze data originating from clinical studies aiming to highlight the possible diagnostic and therapeutic value of ILs on efficiently managing RIF. From an IVF expert’s point of view, the development of new sensitive diagnostic and therapeutic tools is of added value, since RIF patients experience numerous futile IVF attempts coupled with psychological distress and financial burden [30,31,32]. Furthermore, this review aspires to showcase the existing knowledge gaps with regard to the role of ILs in RIF, mapping future scientific goals on both basic and clinical research levels.

## 2. Methodology Employed for Study Selection

A comprehensive review of the literature was performed in PubMed/Medline, Embase, and Cochrane Central databases up to November 2021. Screening strategy included a combination of medical subject headings (MeSH) terms and keywords, including: “cytokines”; “interleukins”; “embryo”; “uterus”; “decidua”; “implantation”; “implantation failure”; “recurrent implantation failure”; “RIF”; “pregnancy failure”; “IVF failure”; “biochemical pregnancy”. Considering inclusion and exclusion criteria for the study selection process, only full-length articles published in internationally peer-reviewed journals and written in English were considered eligible to be included in this review. In order to provide an all-inclusive analysis of the literature, no other specific inclusion and exclusion criteria were set. Thus, all the types of original studies were evaluated, emphasizing studies performed in humans. Considering the type of the included studies, no specific restrictions were employed and data were obtained from basic research and clinical research articles, including retrospective and prospective observational and interventional studies as well as randomized controlled trials. The only prerequisite for a study to be considered eligible for inclusion was the clear description of the results and the methodology employed. Relative findings obtained from other narrative reviews as well as systematic reviews and meta-analyses were also discussed. From the articles retrieved in the first round of search, additional references were identified by manual citation mining.

## 3. The Role of Interleukins in Embryo Implantation and Pregnancy Establishment

### 3.1. Basic Immunological Characteristics of the Endometrial Microenvironment during Implantation and Pregnancy

Embryo implantation constitutes one of the most vital steps for human reproduction [33]. Implantation is the end-point of a complex cellular and molecular dialogue between two distinct components, namely a viable good-quality embryo characterized by a high developmental dynamic and a properly primed and receptive endometrium [34]. One of the most significant categories of molecules regulating this complex dialogue is the several cytokines that are secreted from different types of cells located in the embryo–maternal interface throughout pregnancy. Cytokines are essential for pregnancy establishment playing crucial immunoregulatory roles from decidua and placenta formation till labor induction [4,23,35,36]. More specifically and prior to implantation, the differentiated receptive endometrium, called decidua, is enriched with numerous immune cells and especially with uterine natural killer (uNK) cells, macrophages as well as from a highly differentiated type of cell known as decidual stromal fibroblast cells (DSCs) [26,37,38]. These cells are programmed to secrete pro-inflammatory cytokines and growth factors regulating decidua receptiveness and most importantly maternal resistance to embryo invasion [39]. It seems that cytokines orchestrate an immunological balancing between inflammatory and anti-inflammatory responses and are required for establishing maternal tolerance to the semi-allogenic fetus as well as for protecting both the mother and the fetus from infections throughout pregnancy [40,41]. Thus, many fetal, maternal, and placental mechanisms work simultaneously and synchronously establishing embryo–maternal communication in order to support embryo implantation and pregnancy establishment [42].

Prior to elaborating on the role of ILs in implantation, it is of high significance to map the endometrial immune environment, namely, the immune cell types that play a critical role in implantation and pregnancy [43]. It has been demonstrated that leukocytes account for 30–40% of all decidual cells during the early stages of pregnancy and remain present throughout pregnancy [44,45]. The majority of the leukocyte population consists of T lymphocytes or T cells, uNK cells, and macrophages [26,45,46]. The population of T cells composes 45–60% of the total leukocyte population in the early proliferative endometrium [47]. They are categorized into CD4+ T cells, which mainly include the subsets of Th1, Th2, Th17, and regulatory CD4+ T (Treg) cells, and cytotoxic T lymphocytes (CTLs), namely, CD8+ T cells [48]. Both Th1 and Th2 cell subtypes secrete several cytokines, which regulate the immune functions in the maternal-fetal interface and act as paracrine immune response modulators [37,49]. According to their pattern of paracrine actions, cytokines can be characterized as pro-inflammatory Th1-type and anti-inflammatory Th2-type [37,49,50]. The pro-inflammatory Th1 cell subtype secretes cytokines such as interferon-gamma (IFN-γ), tumor necrosis factor-alpha (TNF-α), and interleukins IL-1, 2, 12, 15, and 18, whereas the Th2 subtype secrets anti-inflammatory cytokines, namely IL-4, 5, 6, 10, and 13 as well as granulocyte-macrophage colony-stimulating factor (GM-CSF) [26,46,48,49]. Moreover, uNK cells stand as a category of NK cells that reside in the uterus and dominate in the secretory endometrium as well as during early pregnancy decidua [51,52]. Besides the cytolytic nature characterizing uNK cells, these cells also possess an additional function being a source of cytokines in the endometrium [43,45,52]. Lastly, the macrophages participate in trophoblast invasion, tissue and vascular remodeling, and are divided into activated and alternatively activated macrophages. This categorization is based on the type of cytokines these cells produce. The activated macrophages secrete pro-inflammatory cytokines, whereas the alternatively activated macrophages secrete anti-inflammatory cytokines, respectively [53].

Both successful implantation and pregnancy maintenance are strongly associated with endometrial immune system regulation, which in turn is achieved by the synchronized and balanced secretion of specific types of cytokines. This unique phenomenon is characterized by periodical changes in the dominant type of secreted cytokines, namely the Th1/Th2 ratio, as significant changes are observed during the different stages of the pregnancy [54,55]. More specifically, during the implantation period and at the early stages of pregnancy, an inflammatory environment is established in the decidua promoting successful embryo implantation and adequate placentation [56,57]. In contrast to general belief, production of pro-inflammatory Th1 cytokines during this phase is required for the establishment of a healthy pregnancy, since this inflammatory microenvironment promotes significant actions, including embryo-maternal immune tolerance, extravillous trophoblast invasion, and spiral arteries remodeling [57,58]. Therefore, a shift towards the Th1 immune response is detected over the Th2 immune response throughout the peri-implantation period, mediating implantation and placenta formation [55]. Afterwards, during the second phase of a pregnancy, an anti-inflammatory Th2 state predominates promoting normal embryo growth and development, as well as protecting both the mother and the fetus from possible infections [55,57]. Indeed, the Th milieu during this period of pregnancy is moved towards the Th2 immune response as Th1 immunity is suppressed [55,57,58]. Lastly, labor induction is associated with a renewed inflammation that requires a pro-inflammatory microenvironment promoting uterine contraction, infant delivery, and placenta rejection [57,59]. Consequently, the Th1/Th2 ratio fluctuates during the different pregnancy stages, reaching a peak in the proliferative endometrium, then, dropping during the following stages, and finally, rising again during labor induction [57,58].

In conclusion, the balanced expression and secretion of Th1 and Th2 cytokines are pivotal for implantation, pregnancy establishment, pregnancy maintenance, placenta formation, and embryo development. Thus, dysregulations in the endometrial immune system profile, mainly originating from abnormal balancing between Th1/Th2 cytokines, are considered to be at the basis of several pathological conditions and adverse events during pregnancy. These include implantation failure, recurrent miscarriages, preeclampsia, and abnormal placentation as well as preterm delivery and compromised embryo development [55,60,61,62].

### 3.2. A Summary of Molecular Actions of Interleukins during Embryo Implantation

It is well documented that cytokines and especially ILs constitute master regulators of the implantation process, mediating significant actions including embryo–maternal communication and synchronization, fostering tolerance, tissue invasion, spiral artery remodeling, and finally placenta formation. In order to better understand the molecular basis of RIF, it is of value to highlight the most important types of ILs playing crucial roles during implantation and to elaborate on the molecular mechanisms via which these ILs regulate complex molecular and cellular networks finally leading to implantation.

#### 3.2.1. The Role of Interleukins with Anti-Inflammatory Properties

The members of the IL-6 family and especially IL-6, IL-11, IL-27 as well as the leukemia inhibitory factor (LIF) act during several stages of pregnancy mediating important anti-inflammatory actions [63,64,65,66,67]. Their common characteristic is that their actions are mediated mainly via the glycoprotein 130 (gp130) receptor signaling [63,64,65,66,67]. Accumulating evidence suggests that among IL-6 family members, LIF presents with the most important properties during implantation [68,69,70,71].

**Figure 1:**
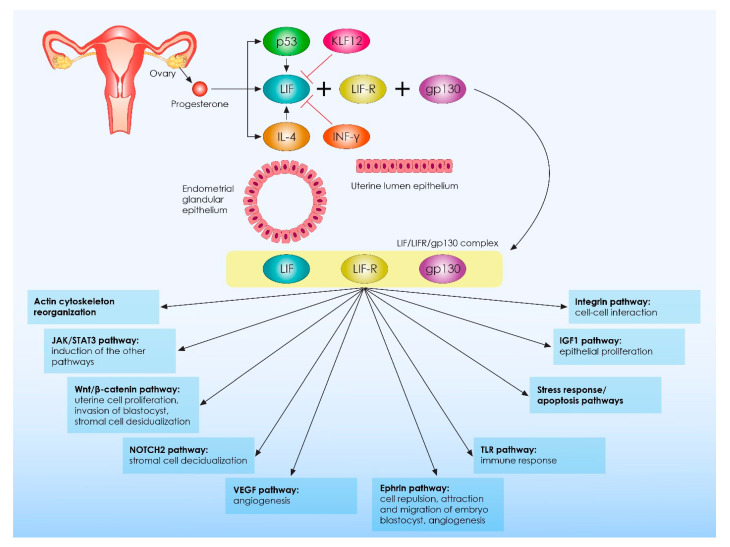
Molecular action of leukemia inhibitory factor during embryo implantation and pregnancy establishment. LIF: leukemia inhibitory factor; KLF12: Krüppel-like factor 12; INF-γ: interferon-gamma; LIF-R: leukemia inhibitory factor receptor; gp130: glycoprotein 130; IGF1: insulin-like growth factor 1; TLR: toll-like receptor; VEGF: vascular endothelial growth factor; Wnt: Wingless/Int; JAK: Janus kinases; STAT3: signal transducer and activator of transcription factor 3.

With regards to LIF characteristics, it is a pleiotropic anti-inflammatory cytokine mostly expressed throughout the mid-to-late luteal phase of the menstrual cycle as well as during the early stages of pregnancy. Considering LIF expression patterns, it is detectable in both the glandular and luminal epithelium [64,68,72]. Its expression is upregulated by IL-4 and progesterone, and it is mainly downregulated by IFN-γ [64]. Moreover, abnormally increased levels of endometrial Krüppel-like factor 12 (KLF12), that have been observed in RIF cases, could also repress LIF expression leading to abnormal decidualization and embryo adhesion and thus to implantation failure [69]. Considering LIF actions, this molecule serves as a regulator of several molecular and cellular processes during implantation via binding to the membrane-bound LIF receptor (LIFR) and gp130. Elaborating on its molecular nature, it is demonstrated that LIF presents in three spliced forms, namely membrane-associated, diffusible, and truncated forms serving as paracrine factors [68]. Following binding to LIFR, LIF recruits gp130 to form a highly effective receptor complex, which in turn activates several downstream signaling pathways, namely the STAT pathway as well as the Janus kinases (JAK) pathway [71,73]. The main final end-point of these intracellular processes is the phosphorylation and the subsequent activation of the STAT3 transcription factor, which mediates significant actions required for proper implantation. The JAK-STAT pathway is mainly inhibited by the suppressor of cytokine signaling 3 (SOCS3). Thus, SOCS3 regulates LIF actions during decidualization, embryo implantation as well as during the early stages of pregnancy establishment via a negative-feedback loop [64,71,74,75,76,77]. Besides JAK/STAT pathway activation, LIF promotes the activation of more than 15 pathways, including the insulin-like growth factor 1 (IGF-1) signaling pathway, the vascular endothelial growth factor (VEGF) pathway, the toll-like receptors (TLRs) pathways, the ephrin pathway, Notch2 signaling, the canonical transforming growth factor (TGF)-β, and the Wingless/Int (WNT) pathway. Moreover, LIF regulates integrin expression as well as stress and apoptosis response pathways. Considering the aforementioned as well as the fact that the great majority of LIF’s regulating pathways play crucial roles in immune system regulation, we can safely conclude that LIF should be considered as one of the master regulators of immune responses observed during the peri-implantation period [78,79]. Considering LIF’s biological role, it has been found that LIF regulates the actions of a vast majority of immune cells that are present in the endometrium during implantation and it also controls the interactions between the decidual immune cells and trophoblast cells of the blastocyst [63]. Generally, LIF’s molecular actions are important for several biological processes taking place during implantation, including endometrial leukocyte recruitment, decidualization, and uterine transformation into a receptive microenvironment as well as for embryo–endometrial interactions and trophoblast invasion [71]. Interestingly, LIF also supports embryo development, an action mainly mediated via the hormone Leptin. It has been voiced that Leptin, acting via the LIF pathway, increases blastocyst formation and blastocyst hatching rates, while it inhibits cell apoptosis during the early stages of embryo development via the STAT3 signaling pathway [80]. A summary of LIF’s molecular action is presented in Figure 1.

**Figure 2:**
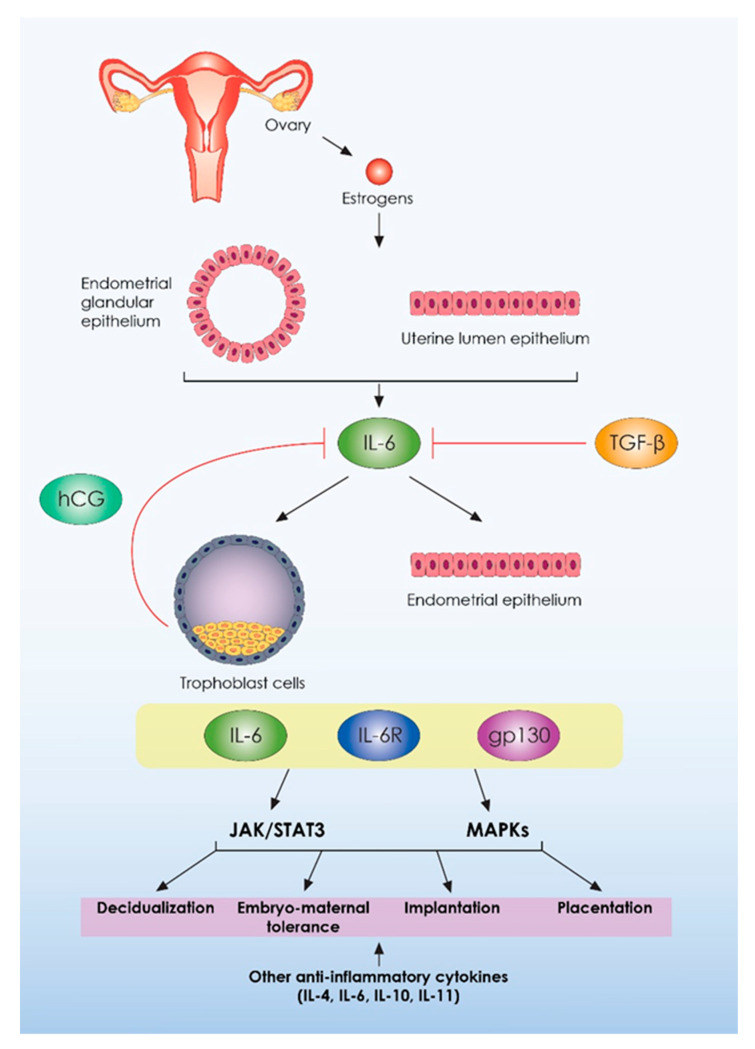
Molecular action of IL-6 and other anti-inflammatory cytokines during embryo implantation and pregnancy establishment. IL-6: interleukin 6; hCG: human chorionic gonadotropin; IL-6R: interleukin 6 receptor; TGF-β: transforming growth factor-beta; JAK: Janus kinases; STAT3: signal transducers and activators of transcription; MAPKs: mitogen-activated protein kinases.

Another important pleiotropic cytokine, which acts as both pro-inflammatory cytokine and anti-inflammatory myokine and presents similar actions to LIF, is the IL-6. Interleukin-6 is secreted from the glandular and luminal epithelial cells of the endometrium throughout the menstrual cycle, especially in the mid-secretory phase as well as during the early stages of pregnancy [81,82]. Like LIF, IL-6 acts via gp130 following the activation of its specific IL-6 receptor (IL-6R). The basic downstream signaling pathways mediating IL-6 actions are the STAT3 pathway as well as the mitogen-activated protein kinases (MAPKs) pathway [64,82]. Receptors for this IL have been found in both human endometrium and trophoblast cells, underlying its significance during implantation and embryo-maternal communication [63,83]. Regarding IL-6 interaction with other hormones and factors, it has been reported that estrogens activate IL-6, but human chorionic gonadotrophin (hCG) and TGF-β inhibit its expression [63]. In fact, hCG, which principally constitutes the first molecule secreted by the early embryo, binds to its specific receptor, namely the luteinizing hormone/choriogonadotropin receptor (LHCGR), inducing LIF production and inhibiting IL-6 secretion by endometrial cells [83,84]. These data demonstrate that the embryo per se is able to control the molecular events involved in its implantation, adjusting a balanced expression of two factors promoting these events, namely LIF and IL-6 expression patterns. Except for LIF and IL-6, there are also plenty of other anti-inflammatory interleukins that play crucial roles during decidualization, implantation, placentation as well as during the earlier and later stages of pregnancy, namely IL-11, IL-4, IL-5, IL-6, IL-9, IL-10, IL-13 as well as IL-27 [85,86]. Among these interleukins, the role of IL-4, IL-6, IL-10, and IL-11 should be emphasized. These molecules principally serve as regulators of embryo-maternal tolerance and their expression is required for establishing a tolerant immunological milieu in the endometrium as recent research indicates [86]. A summary of IL-6 and of other anti-inflammatory cytokines’ molecular action is presented in Figure 2.

#### 3.2.2. The Role of Interleukins with Pro-Inflammatory Properties

During implantation, a unique phenomenon is observed. Successful implantation does not only require the secretion of anti-inflammatory cytokines, but also the production of pro-inflammatory cytokines. This is a paradox considering that in the same biological process, namely implantation, two antagonistic immune phenomena are taking place at the same time.

Concerning the main representatives of pro-inflammatory cytokines, the role of IL-1β should be analyzed [76]. Interleukin-1 beta (IL-1β) is a member of the IL-1 family and it presents with pro-inflammatory properties. It is principally expressed at high levels by cytotrophoblast cells during the first trimester of pregnancy and then, its secretion is decreased [76,87]. Data have shown that IL-1β can also activate the expression of matrix metalloproteinase-9 of cytotrophoblasts, which is an essential molecule mediating trophoblast invasion. Moreover, IL-1β also promotes the expression of B3 integrin, standing as a well-established implantation marker [88]. There is also strong evidence suggesting that IL-1β has a leading biological role during decidualization, as it induces the expression of cyclooxygenase-2 (COX2) and prostaglandin E2 (PGE2). In turn, COX2 and PGE2 represent critical molecules increasing the levels of cAMP in endometrial stromal cells, as cAMP is necessary for proper decidualization [76,89]. Moreover, it seems that IL-1β controls the activity of progesterone throughout pregnancy, although no safe conclusions can be drawn with regard to this observation, since some studies have proven the inhibitory effect of IL-1β on progesterone, whereas others indicate stimulating effects [88,90,91]. Considering the aforementioned, it seems that the actions of pro-inflammatory cytokines, such as IL-1β, are required for establishing an inflammatory environment during implantation, which in turn enhances trophoblast invasion and spiral artery remodeling.

**Figure 3:**
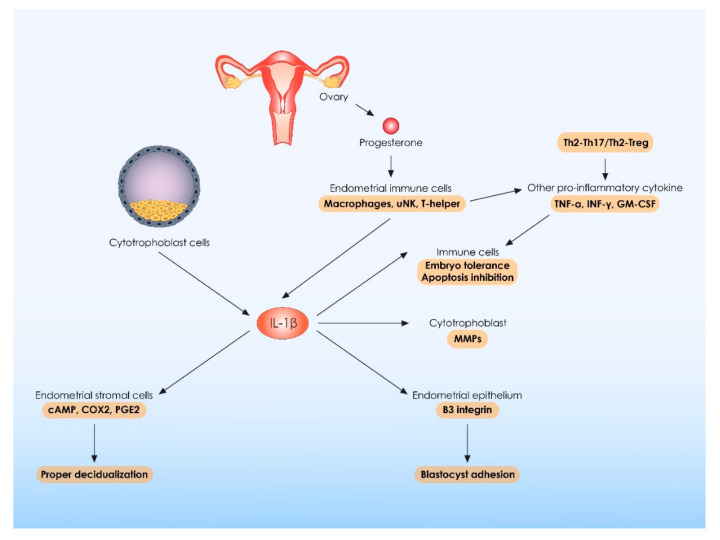
Molecular action of IL-1β and other pro-inflammatory cytokines during embryo implantation and pregnancy establishment. IL-1β: interleukin 1 beta; uNK: uterine natural killer cells; MMPs: matrix metalloproteinases; cAMP: cyclic adenosine monophosphate; COX2: cyclooxygenase-2; PGE2: prostaglandin E2; TNF-α: tumor necrosis factor-alpha; INF-γ: interferon-gamma; GM-CSF: granulocyte-macrophage colony-stimulating factor; Th: T helper cells; Treg: T regulatory cells.

Except for IL-1β, there are several other pro-inflammatory cytokines, which are required for proper implantation, namely INF-γ, TNF-α, IL-12, and IL-17. The original hypothesis was that the presence of these molecules could detrimentally affect embryo implantation, principally leading to embryo rejection. This hypothesis was based on observations supporting that the Th1-type cytokines, namely TNF-α and IFN-γ as well as the Th17 cytokine IL-17, present with embryotoxic and anti-trophoblastic properties [86]. However, the truth is that strictly regulated levels of pro-inflammatory cytokines are required during the peri-implantation period, as indicated by evidence suggesting that the abnormal downregulation of these cytokines could lead to abnormal implantation and trophoblast invasion and subsequently to abnormal placentation and thus to pregnancy failure [55]. In order to better understand these confusing data, it would be of interest to elaborate on the functions and the molecular actions of IL-17 and IL-22, which are both pro-inflammatory cytokines, crucial for embryo implantation [86]. It is evident that IL-17 and IL-22, which are both produced by T helper cells as well as from placental macrophages and trophoblast cells, could at the same time positively and negatively impact the implantation process [86,92,93,94,95]. The final outcome is strongly related to the Th17/Treg ratio as well as to the possible co-expression of the anti-inflammatory cytokine IL-4 [86,96,97,98]. Particularly, on the grounds of pregnancy failure, an increase in the numbers of IL-17-producing CD4+ T cells has been observed, with a subsequent decrease in the number of Tregs. Under these circumstances, uncontrolled IL-17 secretion stimulates embryotoxic phenomena leading to rejection of paternal HLA-C, which is expressed in trophoblast cells. Moreover, the embryotoxic actions of IL-17 are enchased via the action of other pro-inflammatory cytokines, such as TNF-α, IL-1β, IFN-γ, GM-CSF, and IL-22 [55,86,99]. On the other hand, when an increased activity of Th2-Th17/Th2-Treg shift is observed, with a parallel decreased activity of Th1-Th17/Th1 shift, IL-17 promotes embryo implantation and proliferation and inhibits apoptosis of human trophoblastic cells. Under normal conditions, these beneficial effects of IL-17 are mainly mediated by the co-secretion of the anti-inflammatory cytokine IL-4 [86,100,101]. These interesting findings support a new hypothesis with regards to the role of pro-inflammatory cytokines during implantation. It seems that the expression of these potentially harmful molecules is an absolute requirement for normal implantation and their effects are beneficial when a balanced Th1/Th2/Th17 and Treg microenvironment is established during the peri-implantation period. A summary of IL-1β and other pro-inflammatory cytokines’ molecular action is presented in Figure 3.

In summary, a balanced secretion of both anti-inflammatory and pro-inflammatory cytokines is required for proper embryo implantation and pregnancy maintenance. These distinct immunoregulatory elements support different but equally significant biological actions. On one hand, anti-inflammatory cytokines, such as LIF and IL-6, play crucial roles during decidualization and endometrial transformation to a receptive state, while also serving as significant regulators of embryo-maternal communication. Their expression is also required for establishing a tolerant immunological milieu in the endometrium. Moreover, these molecules mediate significant actions during all stages of implantation from apposition and adhesion to trophoblast cells invasion and migration into the decidua. Furthermore, anti-inflammatory cytokines support embryo development. On the other hand, pro-inflammatory cytokines are required for endometrial remodeling that takes place during decidualization as well as during trophoblast invasion and spiral artery remodeling. Moreover, these cytokines are required for proper recognition and immunological acceptance of the semi-allogenic embryo. Their pro-inflammatory properties also inhibit apoptotic phenomena of human trophoblastic cells, which are observed during the dynamic changes taking place during embryo implantation and trophoblast invasion. Thus, a controlled increase in pro-inflammatory Th1 shift is normally observed during the peri-implantation period. Taking into account these dynamic observations, the value of clinical studies investigating them and showcasing their role in implantation failure pathogenesis is what will lead to robust conclusions that could be translated in clinical practice when considering RIF.

## 4. Clinical Data Associating Cytokine Profile with Recurrent Implantation Failure

Based on current data, there is a relative paucity of information concerning the importance of ILs in RIF. However, there are a number of studies investigating the uterine milieu and especially, the cytokine endometrial profile on the grounds of RIF [28].

### 4.1. Clinical Data Associating Leukemia Inhibitory Factor (LIF) with Recurrent Implantation Failure

Acknowledging the importance of LIF in implantation, it is of significance to primarily present the current clinical evidence with regard to the role of LIF in RIF pathogenesis. The first data associating alerted LIF secretion with RIF pathogenesis were provided by the study of Hambartsoumian et al., 1998 [102]. In this study, an in vitro analysis of LIF secretion in explant endometrial cultures was performed, employing enzyme-linked immunosorbent assay (ELISA). Endometrial tissue samples were collected from both the proliferative and the secretory phase employing 32 women with unexplained infertility and/or multiple implantation failures as well as from 17 fertile women, who served as the control group [102]. Leukemia inhibitory factor levels were recorded to be 3.5 times higher in the control group in comparison to the group of patients presenting with unexplained infertility and multiple implantation failures [102]. Moreover, a dysregulation of LIF production in the endometrium was observed in the great majority of infertile women during both the proliferative and secretory phases of the menstrual cycle. The authors concluded that the dysregulation of endometrial LIF secretion throughout the menstrual cycle may be a possible cause of unexplained infertility and recurrent implantation failure [102]. Another interesting study was conducted by Steck and colleagues in 2004, where the prevalence of LIF gene mutations in a total of 47 women with a history of RIF was investigated [103]. Results provided from this study indicate no increased prevalence of functional mutations in the LIF gene in the RIF group in comparison to fertile controls. Thus, we can assume that LIF alterations observed in RIF patients are probably not associated with errors in LIF per se, but in contrast, they may be attributed to impaired synergetic LIF actions with other immunoregulatory molecules in the maternal–fetal interface. At present, dysregulations have been reported in several major LIF-related signaling pathways and genes critical for LIF action. For example, in the study published by Choi and colleagues, an integrative analysis of uterine transcriptome and MicroRNAome was performed, indicating compromised LIF-STAT3 signaling and progesterone response in endometrial samples obtained from RIF patients [104]. Another recently published well-designed study provides evidence regarding a possible molecular mechanism explaining the observed downregulation of LIF in RIF pathogenesis [69]. Results of this study indicate abnormally increased levels of KLF12 in women with a history of RIF, coupled with abnormally decreased levels of LIF. Moreover, provided data indicate that KLF12 inhibits embryo adhesion both in vivo and in vitro by repressing LIF expression. Following analysis of the LIF gene sequence, the authors revealed that KL12 can directly suppress LIF expression, as it can bind to two KLF12-binding sites in the promoter of the LIF gene. Interestingly, the authors also proved that KLF12 expression could be significantly reduced in vitro, when Ishikawa cells overexpressing KLF12 are treated with medroxyprogesterone acetate (MPA). These findings suggest that progesterone may be a novel therapeutic regimen for patients with RIF, as it acts upstream of both LIF and KLF12, inhibiting KLF12 expression and promoting LIF secretion [69].

### 4.2. Clinical Data Associating Anti and Pro Inflammatory Cytokines with Recurrent Implantation Failure

Considering the aforementioned, it appears that LIF plays crucial roles in RIF pathogenesis. However, to better understand RIF pathophysiology, it is important to elaborate on the possible role of the other anti- and pro-inflammatory cytokines presented in the previous chapter of this review. On that account, a prospective study was conducted in 2003 analyzing the intra-uterine cytokine concentration and matrix-metalloproteinase activity in women with unexplained recurrent failed embryo transfers [105]. Briefly, 22 women, who did not achieve ongoing pregnancy following the transfer of 10 or more embryos, were subjected to uterine cavity irrigation during the mid-luteal phase of the menstrual cycle. Irrigation was achieved employing sterile saline infusion into the uterine cavity via a flexible infant feeding tube. The uterine contents were aspired and the levels of TNF-α, IFN-γ, LIF, IL-10 as well as the activity of MMP-2 and MMP-9 were analyzed via ELISA and gelatin zymography, respectively. As controls, 16 samples were also collected from multiparous women with a medical history of tubal sterilization who underwent surgical tubal anastomosis as well as from 13 women who presented with three or more consecutive spontaneous abortions. The pattern of cytokine profile that was discovered showed that in the recurrent embryo transfer failure group, the levels of TNF-α and IL-1β were higher, whereas the concentrations of IFN-γ, IL-10, and LIF were lower compared to those observed in control groups. Further to that, the MMP activation score was found to be elevated in the recurrent embryo transfer failure group. This finding was associated with the elevated levels of IL-1β observed in the recurrent embryo failure group, supporting the hypothesis that IL-1β promotes MMPs’ expression and activity [88,105].

Important information, regarding the possible role of IL-1β and TNF-a on the pathophysiology of RIF, is provided from the prospective observational study of Boomsma et al., 2009 [106]. The authors of this study performed a multivariable logistic regression analysis in order to investigate whether the cytokine profiling of endometrial secretions collected immediately prior to embryo transfer in IVF cycles could be indicative of pregnancy outcome. Analysis was performed in 210 women undergoing IVF treatment and none of these patients had undergone more than one prior unsuccessful embryo transfer [106]. In concordance with the study of Inagaki et al., 2003, a significant negative association was observed with IL-1β levels and pregnancy outcome [105,106]. However, and in contrast with the findings of Inagaki et al., 2003, the TNFa levels were strongly positively associated with IVF cycle success [105,106]. These controversial findings with regards to TNF-a could be explained considering the differences among the design of these studies, and more specifically considering the larger population recruited in the study of Boomsma et al., 2009 as well as the different criteria employed for defining the study and the controls groups.

Results provided from a recently published study by Amjadi and colleagues add another level of complexity to the aforementioned [20]. In this study, an innate and adaptive immune system polymerase chain reaction (PCR) array was used to compare endometrial transcriptomic profiles obtained from 11 women with a history of unexplained RIF in comparison to 10 fertile women [20]. In this study, RIF was defined as the failure to achieve pregnancy following the transfer of at least four good-quality embryos in a minimum of three cycles. Statistical analysis revealed higher expression levels of IL-6, INF-γ, IL-2, IL-17A, IL-23, and STAT3, but lower expression levels of IL-1β, IL-8, and NFkB in the RIF group compared to the control group [20]. In addition, increased activation of pNK cells and upregulation of Th17 and TLR signaling pathways were observed in the RIF group. The authors conclude that modulation of the immune system in RIF patients is shifted to inflammatory responses [20]. However, results provided by a similar study conducted by Rajaei and colleagues do not fully support the findings of Amjadi et al., 2020 [36]. In this study, comparisons were made between 10 women with a history of RIF, who were under 40 years of age with three unsuccessful IVF attempts following the transfer of good-quality embryos, and 12 normal fertile women with at least one live birth presenting with no history of infertility [36]. In concordance with the study of Amjadi et al., 2020, lower IL-8 levels were recorded in the RIF group. However, and in contrast to the study of Amjadi, IL-6 levels were lower and IL-10 levels were higher in the endometrial stromal cells of RIF cases compared to the control samples [36]. Summarizing these data, it seems that RIF is probably associated with an abnormal extensive inflammation during the peri-implantation period; however, no safe conclusions can be drawn with regard to the role of the aforementioned ILs on RIF pathophysiology. Discrepancies are observed among the studies, as the same IL in some of these is recorded to be increased in RIF cases, but in some others, it is recorded to be reduced.

### 4.3. Clinical Data Associating Interleukins 12, 15 and 18 with Recurrent Implantation Failure

On another note, the possible implication of IL-15, IL-18, and IL-12 on RIF pathophysiology has been investigated. With regards to IL-15, it is considered to be an essential pro-inflammatory cytokine, having a pivotal role in uNK cell regulation. More specifically, there is evidence suggesting that IL-15 regulates the postovulatory restitution of peripheral blood NK cells into the endometrium [107,108]. The significant role of this cytokine in implantation is highlighted by the fact that this IL is mostly expressed in the endometrium during the secretory phase of the menstrual cycle. In fact, it has been voiced that IL-15 levels are strongly associated with the number of uNK cells and its expression seems to be increased in RIF cases, in contrast to LIF levels which have been reported to be reduced [109]. Regarding IL-18, it seems to be another key element in the maternal–fetal interface, regulating Th1 or Th2 immune responses according to the established immunological microenvironment [110,111]. Pondering on its multifactorial role, IL-18 appears to promote the expression of TNF-α, IL-6, IL-8, and IL-1β [111]. Notably, it has been proven that it can act synergistically with IL-15 regulating uNK proliferation in vitro [112]. In addition, IL-12 constitutes a pro-inflammatory cytokine that also regulates the activation of uNK cells, while also promotes the secretion of IFN-γ in high doses [113,114,115].

The first evidence indicating a possible implication of IL-12 and IL-18 in RIF pathology, is provided by the controlled clinical study of Lédée-Bataille et al., 2004 [114]. The aim of this study was to evaluate a possible association between endometrial immunohistochemical staining of interleukin IL-12 and IL-18 with the number of CD56^bright^ NK cells as well as with Doppler vascular disorders, in RIF patients. For the purpose of this study, 35 women with a history of RIF and 12 fertile women serving as the control group, were subjected to ultrasound evaluation and endometrial biopsies on day 20 of the menstrual cycle. Considering the findings of this study, RIF patients presented with an increased CD56^bright^ NK cell number, strong anti-IL12 and IL-18 staining, and an increased proportion of abnormal vascular parameters [114]. These data suggest that the altered expression of IL-12 and IL-18 could not only negatively affect the immune uterine microenvironment, but could also jeopardize proper endometrial vascularization, detrimentally affecting endometrial receptivity and subsequently implantation [114]. Following these interesting findings, several other studies were conducted indicating that alterations in the expression patterns of the triplet IL-12/Il-15/IL-18 could lead to abnormal uNK hyperactivation coupled with abnormal endometrial vascularization [19,116,117,118,119,120,121,122,123]. Considering the molecular mechanism via which the abnormal expression of the triplet IL-12/Il-15/IL-18 could lead to abnormal endometrial vascularization, there are data suggesting that these ILs regulate the expression of endometrial tumor necrosis factor-like weak inducer of apoptosis (TWEAK) and its receptor, namely the fibroblast growth factor inducible-14 (Fn-14). Briefly, TWEAK is a member of the TNF superfamily; it is expressed by several immune and endometrial cells and appears to be essential for regulating the cytotoxic nature of uNK cells during implantation. Moreover, TWEAK presents significant angiogenic properties. Thus, alterations in TWEAK expression, originating from abnormal expression of the triplet IL-12/Il-15/IL-18, could lead to both uNK cell cytotoxicity and to abnormal endometrial vascularization and thus to RIF [19,116,117,118,119,120,121,122,123].

### 4.4. Abnormally Increased Levels of Pro-Inflammatory Cytokines Originating from Abnormal Regulation of T and B Regulatory Cell Function Are Associated with Recurrent Implantation Failure

Taking into account that alterations in IL-12/Il-15/IL-18 and TWEAK expression patterns are associated with increased uNK cytotoxicity, one could extrapolate that similar alterations regarding other cytokines, such as IL-10, TNF-a, IL-4, IL-5, IL-10, IL-13, and GM-CSF could lead to abnormally increased numbers of cytotoxic T cell and B cell subtypes [122,124,125,126,127]. These alterations commonly originate from the abnormal functioning of Treg and Breg cells, which in RIF cases tend to overproduce pro-inflammatory cytokines inducing immune embryotoxic phenomena finally resulting in RIF or/and recurrent miscarriages. This cytotoxic function of regulatory immune cells has been reported in both peripheral blood and endometrial tissue samples obtained from RIF patients [122,124,125,126,127]. For example, in the study published by Fukui et al., 2008, the numbers of TNF-a-producing CD56^bright^NK cells was significantly increased in women with RIF compared with fertile controls [126]. Under normal conditions, TNF-a is required for the proper differentiation as well as for the proper development of trophoblast cell, but abnormally increased levels of TNF-a could result in implantation failure and abortion [125,126,128]. In addition, a recently published study by Koushaeian et al., 2019 provides evidence indicating that the downregulation of peripheral blood IL-10-producing Breg cells might result in RIF pathogenesis. Moreover, the authors suggest that the downregulation of IL-10-producing Breg cells maybe result in the production of anti-fetal antibodies by cytotoxic subtypes of B cells; under normal conditions, Breg cells suppress antibody generation, contributing to successful implantation [127,129]. Considering the aforementioned, we can conclude that both Treg and Breg cells are an absolute requirement for establishing normal maternal–fetal interactions, and this is underlined by the fact that RIF is characterized by a decreased number of total effector Treg and Breg cells.

The data that have so far been presented indicate that among RIF cases there is a great heterogeneity with regards to the type of immune system dysregulation leading to implantation failure and this heterogeneity is mainly attributed to the complex actions and interactions of ILs during the peri-implantation period. Moreover, it is demonstrated that alterations in ILs’ expression pattern do not only affect endometrial microenvironment but also could detrimentally affect several other biological mechanisms, including endometrial vascularization.

### 4.5. Alterations in Upstream Pathways Regulating Interleukin Production Are Associated with Recurrent Implantation Failure

However, recently published evidence indicates that alterations in factors acting upstream of ILs in the molecular cascade of implantation could detrimentally affect IL actions subsequently leading to RIF. Thus, it is significant not only to investigate ILs’ endometrial profile in RIF cases but also to investigate whether alterations are attributed to ILs per se or to other factors regulating IL expression patterns. Elaborating on that, data provided from a recently published study indicate a reduction in the expression levels of progesterone-induced blocking factor 1 (PIBF1) in the mid-secretory endometrium in RIF patients compared to a non-RIF infertile group [130]. Under normal conditions, PIBF1 binds to the promoter of IL-6, activating IL-6 expression. In turn, IL-6 activates the phosphorylation of STAT3, which mediates anti-inflammatory phenomena, regulating proper embryo implantation [130,131,132]. Thus, we can assume that PIBF1 is a significant regulator of the implantation process. This hypothesis is supported by in vitro experiments performed in Ishikawa and human endometrial stromal cells (HESCs), indicating that PIBF1 knockdown significantly downregulates IL-6 and p-STAT3 expression and thus cell proliferation and decidualization [130]. Interestingly, progesterone induces PIBF1 expression and both progesterone and PIBF1 suppress decidual lymphocyte cytotoxicity [133]. These findings highlight the possible therapeutic role of progesterone administration in RIF cases characterized by the altered expression of PIBF1.

Focusing further on the genetic background of RIF-related immunological alterations, a knowledge gap is identified as little data are available in the current literature. However, a recently published study provides interesting information with regards to the incidence of specific single nucleotide polymorphisms on p53, IL-11, IL-10, VEGF, and Apolipoprotein E (APOE) genes in women with a history of RIF [134]. In contrast to the expected outcomes, no statistically significant difference was observed in the prevalence of IL-11 and IL-10 mutations in RIF patients [134]. Nonetheless, an increased prevalence was observed among RIF patients in mutations related to p53 and VEGF. These findings are also confirmed by previously conducted studies [135,136,137]. The question is “are these alterations observed in p53 and VEGF genes associated with impaired IL regulation on the grounds of RIF?”. The answer is yes, considering that both p53 and VEGF are essential for embryo implantation. On one hand, p53 induces LIF expression and thus constitutes an important regulator of endometrial receptivity and implantation. On the other hand, VEGF mediates significant angiogenic actions regulating vascular remodeling of the uterus during the peri-implantation period, while more importantly VEGF actions are regulated by LIF [134,138,139]. This is an exceptional second paradigm on how alterations observed in molecules acting upstream to the IL network could detrimentally affect the immunological balance established in the maternal–fetal interface finally leading to RIF. Clinical data associating cytokine profiles with RIF are summarized in Table 1.

## 5. Discussion

**Figure 4:**
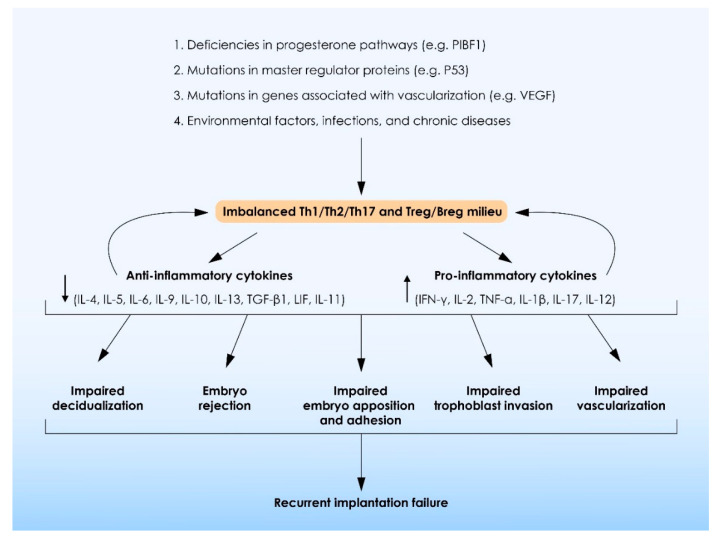
A summary of the role of interleukins on the events entailed in recurrent implantation failure (RIF) pathogenesis.

According to our knowledge, this is the first review of the literature summarizing the current evidence with regards to the role of ILs in the multifactorial condition of RIF. Evidence provided is summarized in Figure 4 and highlight that alterations in expression patterns of ILs in the maternal–fetal interface negatively affect numerous biological processes required for proper embryo implantation and pregnancy establishment. These include: decidualization, immunological acceptance of the semi-allogenic blastocyst, embryo–maternal communication, embryo attachment to the endometrium, trophoblast cell invasion, spiral artery remodeling, embryo development, and several others. Moreover, the present review highlights the significant role of ILs in establishing a balanced Th1/Th2/Th17 and Treg microenvironment during the peri-implantation period, which in turn is required for implantation and proper development of the early-stage embryo. In addition, we herein provide clear evidence indicating that, in contrast to general belief, a controlled increase in pro-inflammatory Th1 shift is required during the peri-implantation period.

Considering the clinical significance of ILs in RIF pathogenesis, the association between ILs and RIF has been extensively investigated in several studies, as indicated in the review of literature herein. However, several discrepancies are observed between the studies, mainly originating from the variant research methodology employed as well as from the lack of a universally accepted definition of RIF, coupled with the lack of a commonly accepted protocol for IL profiling in RIF patients. This highlights the need for further research examining a different perspective. Future studies should focus on recruiting participants employing standardized criteria, in order to reduce the heterogeneity observed among RIF patients. Moreover, there emerges a clear need for the development of an efficient research protocol for studying the microenvironment of the maternal–fetal interface. Newly developed laboratory assays, such as organ-on-a-chip (OOAC) technology could serve as an ally and significant tool towards that goal. Additionally, studies resulting in mere associations and observations may thenceforth be regarded as redundant, and may no longer be of value, especially when there is a clear need for deep analysis leading to robust data regarding the molecular “how, when, and why” ILs that affect embryo implantation. In the “era of omics”, these data could become available. Elaborating on that, the use of “omics” technology could provide a detailed “molecular footprint” of interactions between ILs and other molecules acting upstream or downstream in the molecular cascade of implantation. In turn, a systematic, methodical analysis fulfilling these high analytical specifications could enable a clear and robust profiling of RIF pathogenesis in an individualized manner.

The deep profiling of ILs network in RIF cases does not represent “a research for the sake of research” approach, but in contrast emerges as an absolute requirement for aptly diagnosing the underlined pathological mechanisms leading to RIF, and thus buttressing the development of more efficient, safe, and cost-effective management protocols. Despite the great advances that have been observed in the field of reproductive medicine and reproductive immunology, RIF management remains a conundrum stemming from the inadequate diagnosis of the actual pathogenesis entailed in RIF. Several management protocols have been proposed so far, including endometrial scratching, adjuvant glucocorticoid administration, intravenous immunoglobulin (IVIg) administration, and intralipid therapy. However, none of the aforementioned management protocols have been proven conclusively efficient to the extent of leading to a consensus [16,140,141]. In contrast, clinicians should be aware that the liberal use of these immunoregulatory treatments could not only be inadequate, but in certain circumstances, could even be rendered harmful. For example, the excessive iatrogenic immunosuppression of the Th1 shift during the peri-implantation period could also result in implantation failure. At the same time, IL profiling could efficiently guide clinicians to make evidence-based decisions on the grounds of individualized and precision medicine. For instance, in cases where RIF is caused by alterations in KKLF12 and PIBF1 actions, the treatment may be as simple as progesterone administration. Moreover, IL profiling could be proven beneficial, not only for the management of unexplained RIF cases but also for the management of infertile patients suffering from conditions causing endometrial immune system dysregulations, such as endometriosis, adenomyosis, and chronic endometritis.

This review collectively analyzes the data presenting associations between various ILs and implantation failure, and uniquely brings to the literature the conclusion that the role of ILs in RIF is indeed significant. Further research employing state-of-the-art methodologies at the basic, translational, and clinical level will unveil promising and powerful tools in aptly diagnosing and treating the pathogenesis leading to RIF. Considering that the scientific community is still in search of the “holy grail” in addressing the challenge that RIF represents, studying ILs could emerge as a valuable ally for the researcher and the clinician called to delineate implantation failure and manage the multifactorial condition of RIF.

## Figures and Tables

**Table 1:** Summary of the current evidence regarding the role of interleukins in recurrent implantation failure, highlighting examined parameters as well as major findings of the reviewed studies.

Publication.	Study Design	Study Group(s)	Control Group	Outcome Measures	Conclusion
[134]	Retrospective case control study	-RIF patients -RPL patients	Oocyte donors with no history of RIF or RPL	Prevalence of polymorphisms in p53, VEGF, IL-10, IL-11 and APOE	Correlation of p53 and VEGF polymorphisms with RIF and APOE polymorphisms with RPL.
[20]	Prospective study	RIF patients	Fertile women	Expression of IL-6, IFNG, IL-17A, IL-23A, IFN-A1, IFN-B1, CD40L, CCR-4, CCR-5, CCR-6, CXR-3, CCL-2, IL-2, TLR-4, IRF-3, STAT3, RAG1, IFNA-R1, IL-1B, IL-8, NFKB, HLA-A, HLA-E, CD80, CD40	Activation of pNK cells, Th17 signaling pathway and TLR signaling pathway in RIF patients; shift to inflammatory immune responses
[130]	Prospective study	RIF patients	Women with tubal obstruction or unexplained infertility who achieved a clinical pregnancy after the first embryo transfer	Evaluation of PIBF1, IL-6 and p-STAT3 in mid-secretory endometrium	Decreased PIBF1/IL6/p-STAT3 in RIF group. Decreased PIBF1/IL6/p-STAT3 during the mid-secretory phase inhibits human endometrial stromal cell proliferation and decidualization
[127]	Prospective study	RIF patients with immune system abnormalities	Women with at least one successful pregnancy	Evaluation of expression levels of PD-L1 and IL-10 in peripheral blood; serum levels of several autoantibodies including anti-TPO, anti-TG, ANA, ACA, APA	IL-10 producing B- cells are down-regulated in peripheral blood of patients with RIF
[69]	Prospective study in humans and in vivo experiments in mouse model	RIF patients	Age-matched fertile women	Evaluation of KLF12 and LIF expression patterns; evaluation of embryo adhesion in mouse model	KLF12 inhibits embryo adhesion in vivo and in vitro by repressing LIF expression. Increased expression of KLF12 and decreased expression of LIF in RIF patient endometrium
[19]	Observational cohort study	RIF patients	Women who underwent an endometrial sampling 3 months before their ETs and who all successfully gave birth at the first subsequent attempt of fresh or frozen-thawed ET	Ratio of IL-15/Fn-14 mRNA; biomarker of uNK cell activation/maturation and IL-18/TWEAK mRNA ratio; biomarker of both angiogenesis and the Th1/Th2 balance.	Endometrial immune profiles were dysregulated in RIF patients; mostly over activated
[104]	Prospective study	RIF patients	Women under 40 years old, with regular menstrual cycle, who had at least one normal pregnancy and delivery	Endometrial expression of p-STAT3), ERα and PR	Systematic dysregulation of LIF-JAK-STAT3 pathway in RIF patients
[109]	Retrospective study	RIF patients	Fertile women	Evaluation of IL-15 and LIF levels in peripheral blood and endometrial samples, association with uNK cell number	Altered expression of LIF and IL-15 in the endometrium of RIF patients. Correlation between the uNK cell number and the stromal cell IL-15.
[36]	Prospective study	RIF patients of advanced maternal age (age > 40 years old)	Women with proven fertility and under 40 years old, with at least one live birth and no history of abortion or infertility with regular menstrual cycles	Comparison of cytokine profile (IL-10, TGF-β, IFN-γ, IL-6, IL-8 and IL-17) of whole endometrial cells and endometrial stromal cells between the RIF and the control group	Higher levels of IL-6, IL-8 and TGF-β in the endometrial stromal cells and whole endometrial cells of normal fertile women compared to RIF group. Lower levels of IL-10 in endometrial stromal cells of the control group compared to RIF group.
[122]	Ex vivo model	Cell culture from endometrial biopsies from patients with RPL and RIF who had over expression of IL-18 with TWEAK	Cell culture from endometrial biopsies from women with RPL and women with RIF who had over expression of IL-18 without TWEAK	Ex vivo model to study mRNA expressions of NKp46 (uNK cytotoxic receptor) and TGF-β (regulates uNK cytokine production)	TWEAK is a modulator for prevention of endometrial uNK cytotoxicity
[123]	Prospective study	Unexplained RIF patients	Fertile women	Expression of TWEAK, IL-18, b2-microglobulin (b2 M), ribosomal protein L13A (RPL13A), and TATA box-binding protein (TBP)	High levels of IL-18/TWEAK ratio might lead to high levels of uNK cells but low levels of IL-15/Fn-14 might indicate uNK depletion
[116]	Prospective cohort study	RIF patients	Women undergoing IVF/ICSI treatment with successful implantation	Endometrial secretion analysis for 17 soluble regulators of implantation prior to embryo transfer	Clinical pregnancy was correlated with higher concentrations of TNF-α and lower levels of IL-1β. It is suggested that the ratio of TNF-α and IL-1β may appear as an indicator of endometrial receptivity.
[125]	Sequential study	RIF and RPL patients	Fertile women	Peripheral blood NK cells for NCRs (NKp46, NKp44 and NKp30) and cytokine expression (TNF-α, IFN-c, IL-4, IL-10)	Excessive pro-inflammatory cytokine expression in NK cells in RPL and RIF patients
[119]	Control study	RIF patients	Fertile women	Endometrial IL-18, IL-18BP, IL-15 mRNA expressions and number of CD56+ cells	Ultrasonographical indicators appear to be related to insufficient or excessive uNK recruitment and inadequate endothelial vascular remodeling.
[126]	Prospective cohort study	Patients with recurrent spontaneous abortions and RIF	Fertile women	Cytokine expression of interferon-γ, TNF-a, IL-4, IL-5, IL-10, IL-13, and granulocyte-macrophage colony-stimulating factor (GM-CSF) in NK cells	Natural killer-1 shift in peripheral blood samples of patients with recurrent spontaneous abortions and RIF
[120]	Pilot study	RIF patients	Fertile women	Uterine artery Doppler, count of uterine CD56 bright cells and quantification of IL-12 family, IL-18 system, and IL-15 mRNA	Uterine artery pulsatility index is negatively correlated with the IL-18/actin ratio. IL-18 and IL-15 are involved in the local recruitment and the activation of uNK cells. Correlation of IL-18 with IL-15 and IL-12.
[114]	Controlled clinical study	RIF patients	Fertile women	Evaluation of the balance between IL-12 and IL-18; evaluation of the number of uNK cells; evaluation of the endometrial vascular status	Abnormal vascular parameters among RIF group; higher number of uNK cells and altered IL-12 and IL-18 expression patterns.
[103]	Cohort study	RIF and unexplained infertility patients	Fertile women	Genetic analysis for LIF gene mutations	No functional mutations in LIF gene in women with RIF
[105]	Prospective study	RIF patients	-Multiparous women with a history of tubal sterilization who were undergoing surgical tubal anastomosis -Patients having three or more consecutive spontaneous abortions without a live birth	Matrix metalloproteinase (MMP) score and cytokine concentrations in endometrial fluids	In RIF group, the MMP score and IL-1β concentration were significantly higher than those in the control group, whereas concentrations of IFN-γ and IL-10 were significantly lower
[124]	Prospective study	Women undergoing in vitro fertilization treatment	Fertile women	Percentage of expression of CD69+, HLA-DR+ and CD11b+ on CD4 and CD8 T cells and association with implantation failure incidence	T-cell activation markers CD 69+ and HLA-DR+ are associated with increased implantation failure
[102]	Prospective blinded clinical and biochemical study	Women with primary or secondary infertility of unexplained etiology and RIF	Fertile women	Evaluation of LIF secretion in endometrial cultures	Decreased LIF production in endometrial samples obtained from RIF patients

RIF: recurrent implantation failure; RPL: recurrent pregnancy loss; VEGF: vascular endothelial growth factor; IL: interleukin; APOE: apolipoprotein E; IFNG: interferon-gamma; IFN: interferon; CD40L: CD40 ligand; CCR, CXR, CCL: members of chemokine subfamilies; TLR: toll-like receptor; IRF: interferon regulatory factors; RAG1: recombination activating gene 1; IFNA: interferon-alpha; HLA: human leukocyte antigen; PIBF1: progesterone-induced blocking factor 1; p-STAT3: phosphorylated signal transducer and activator of transcription 3; PD-L1: programmed death-ligand 1; anti-TPO: thyroid peroxidase autoantibodies; anti-TG: thyroglobulin autoantibodies; ANA: antinuclear antibodies; ACA: anticentromere antibodies; APA: antiphospholipid antibodies; KLF12: Krüppel-like factor 12; LIF: leukemia inhibitory factor; ET: embryo transfer; Fn-14: fibroblast growth factor inducible–14, TWEAK: tumor necrosis factor-like weak inducer of apoptosis; ERα: estrogen receptor alpha; PR: progesterone receptor; uNK: uterine natural killer cells; TGF-β: transforming growth factor-beta; INF-γ: interferon-gamma; NCRs: natural cytotoxicity receptors; TNF-α: tumor necrosis factor-alpha; IL-18BP: interleukin-18 binding protein; mRNA: messenger RNA; HLA-DR: major histocompatibility complex II cell surface receptor.

## Data Availability

Not applicable.
